# Molecular characterization, tissue expression and sequence variability of the barramundi (*Lates calcarifer*) myostatin gene

**DOI:** 10.1186/1471-2164-9-82

**Published:** 2008-02-19

**Authors:** Christian De Santis, Brad S Evans, Carolyn Smith-Keune, Dean R Jerry

**Affiliations:** 1 Aquaculture Genetics Research Program, School of Marine and Tropical Biology, James Cook University, Townsville, Queensland, 4811, Australia

## Abstract

**Background:**

Myostatin (MSTN) is a member of the transforming growth factor-β superfamily that negatively regulates growth of skeletal muscle tissue. The gene encoding for the MSTN peptide is a consolidate candidate for the enhancement of productivity in terrestrial livestock. This gene potentially represents an important target for growth improvement of cultured finfish.

**Results:**

Here we report molecular characterization, tissue expression and sequence variability of the barramundi (*Lates calcarifer*) *MSTN-1 *gene. The barramundi *MSTN-1 *was encoded by three exons 379, 371 and 381 bp in length and translated into a 376-amino acid peptide. Intron 1 and 2 were 412 and 819 bp in length and presented typical GT...AG splicing sites. The upstream region contained *cis*-regulatory elements such as TATA-box and E-boxes. A first assessment of sequence variability suggested that higher mutation rates are found in the 5' flanking region with several SNP's present in this species. A putative micro RNA target site has also been observed in the 3'UTR (untranslated region) and is highly conserved across teleost fish. The deduced amino acid sequence was conserved across vertebrates and exhibited characteristic conserved putative functional residues including a cleavage motif of proteolysis (RXXR), nine cysteines and two glycosilation sites. A qualitative analysis of the barramundi *MSTN-1 *expression pattern revealed that, in adult fish, transcripts are differentially expressed in various tissues other than skeletal muscles including gill, heart, kidney, intestine, liver, spleen, eye, gonad and brain.

**Conclusion:**

Our findings provide valuable insights such as sequence variation and genomic information which will aid the further investigation of the barramundi *MSTN-1 *gene in association with growth. The finding for the first time in finfish *MSTN *of a miRNA target site in the 3'UTR provides an opportunity for the identification of regulatory mutations on the expression of this gene.

## Background

The transforming growth factor-β (TGF-β) superfamily encompasses a number of peptides, such as inhibins and activins, which share a similar structure and a relatively conserved amino acid sequence. Transforming growth factors are involved in important biological functions, including cell growth and differentiation [1]. Myostatin (MSTN), formerly known as growth differentiation factor-8, is a member of the TGF-β superfamily recently isolated from murine muscle tissue by McPherron *et al*. [2]. The biological activity model proposed for MSTN is experimentally supported by gene inactivation and revealed a negative correlation between *MSTN *expression and growth/number of muscle fibres [2]. The lack of evident non-specific defects in null-*MSTN *mice suggests that mammalian MSTN physiological functions are essentially confined to skeletal muscle. To corroborate this hypothesis, the *MSTN *gene is found expressed almost exclusively in skeletal muscle and heart of higher vertebrates [2,3]. Alternatively, piscine *MSTN *transcripts have been ubiquitously detected in tissues such as muscle, gill, brain, kidney and gonad, highlighting that in lower vertebrates MSTN is possibly involved in several developmental and physiological functions [4,5]. However, MSTN activity has been successfully inhibited in model fish species via N-terminal pro-peptide over-expression, morpholino, or double strand RNA interference, resulting in some degree of hypertrophic and/or hyperplasic muscle mass increase. This suggests that regulation of skeletal growth has remained the principal developmental role of *MSTN *across all vertebrates [6-8].

The *MSTN *gene has been cloned and characterized in a large number of high value commercial fish species such as atlantic salmon [9], shi drum [10], rainbow trout [11], gilthead sea bream [12], white bass [13], Mozambique tilapia [13], catfish spp. [14-16], European sea bass [17], striped bass [18], white perch [18], orange spotted grouper [19], Japanese sea perch [20] and croceine croaker [21]. In fish, this gene consists of three exons and two introns. Exon 1, encoding for the N-terminal signal sequence for secretion, contains the highest inter-specific variability, while exons 2 and 3 are highly conserved across species and are translated into the pro-peptide and C-terminal bioactive dimer [15,21]. Alternative forms of *MSTN *have been independently isolated in zebrafish, gilthead sea bream, fugu and salmonid spp. [9,11,22,23]. Different rates of identity shared between alternative *MSTN *isoforms suggests that at least two events of duplication occurred in finfish. A first event, which separated *MSTN-1 *and *MSTN-2*, occurred early during teleost evolution, while a second event, likely due to tetraploidization, occurred in salmonids (*MSTN1a-2a*; *MSTN1b-2b*) [24]. According to Rodgers *et al*. (2007), the new nomenclature proposed for the *MSTN *gene family has been adopted in this study.

The desire to select fast growing strains for the rapid improvement of livestock production in selective breeding programs has encouraged the identification of genes with a putative role in the enhancement of growth. Several authors have attempted to establish a quantitative correlation between production trait variability and genetic markers. For example, a single nucleotide polymorphism (SNP) occurring at intron 3 of the chicken growth hormone gene was associated with a 16.8% enhancement of body weight [25], and the "long allele" of a microsatellite described at the cattle *g*rowth hormone receptor promoter corresponded to a 6.9% production improvement compared to the "short allele" [26]. Therefore, targeting the improvement of a trait under quantitative genetic control with a single-gene approach (marker assisted selection) ensures more predictable and consistent outcomes compared to widely used traditional breeding techniques, reflecting an increasing demand for additional genes to be characterized in commercial animal production species.

Because of the unquestionable involvement of MSTN in differentiation and proliferation of muscle fibres, *MSTN *gene is considered a candidate for livestock growth improvement. However, while several authors have studied phenotypic implications of naturally occurring mutations in terrestrial livestock [27-31], only a few studies have described polymorphisms in the piscine *MSTN *gene. Maccatrozzo *et al*. [10] detected variation in a Simple Sequence Repeat (SSR) present at the 3'UTR of shi drum (*Umbrina cirrosa*), whereas Kocabas *et al*. [15] described several synonymous and non-synonymous mutations in the catfish *MSTN *gene, including a length polymorphism of a CAG-microsatellite present at exon 1. Surprisingly, neither microsatellites in non-coding regions (probable source of variation in traits under quantitative genetic control such as growth [32]), nor variation at the exons have been investigated in association with growth traits in finfish thus far.

Barramundi (*Lates calcarifer*) is a high-value aquaculture fish endemic and widely farmed in south-east Asia and Australia and with increasing aquaculture interest in Europe and North America [33,34]. On account of the increasing worldwide aquaculture importance of this species, a growing number of farmers have expressed interest in maximising production through selective breeding. Ongoing experiments carried out in our research group have revealed extremely large size variability in F1 hatchery juveniles grown under identical conditions (Smith-Keune *et al*., *unpublished data*). The possibility that a certain amount of the observed variability might be explained by means of additive effects at the *MSTN *gene encouraged us to commence this investigation. The aims of this study, therefore, were to characterize the *MSTN-1 *gene in barramundi and to understand the gene's tissue-specific expression pattern. The project also aimed to identify possible regions of sequence variability among barramundi individuals that could be tested for their correlation with phenotypic growth in future studies.

## Results

### Sequence characterization and analysis

**Figure 1:**
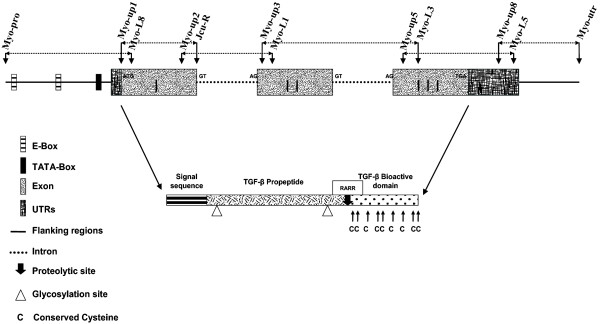
**Schematization of the barramundi *MSTN-1 *gene**. For clarity, the *MSTN *gene and deduced protein have not been represented in their real proportions. Primers sequences are presented in Table 1.

**Figure 2:**
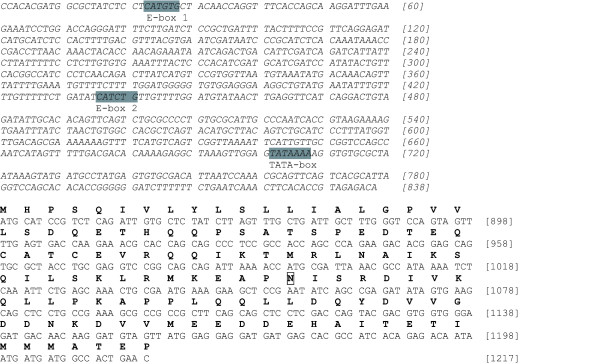
**Genomic sequence of the barramundi *MSTN-1 *gene**. Non-coding regions are represented italicized. TATA-box and E-boxes are grey-highlighted. Coding regions are shown in normal capital letters and deduced amino acid translation is reported in bolded single-letter code. Putative proteolytic and glycosilation sites are boxed and conserved cysteines underlined. Star represents STOP codon. This figure shows the upper quartile, for the full image please see Additional file 1.

PCR amplifications from barramundi tissues resulted in six fragments of the following sizes: 925 bp (Myo-pro/Myo-L8), 384 bp (Myo-up1/Jcu-R), 765 bp (Myo-up2/Myo-L1), 1068 bp (Myo-up3/Myo-L3), 749 bp (Myo-up5/Myo-L5) and 831 bp (Myo-up8/Myo-utr). The relative position of each primer is shown in Fig. 1. The whole consensus sequence (4484 bp) (Fig. 2), including 5' and 3' flanking region, was obtained by merging overlapping PCR fragments and has been deposited in Genbank [35] (accession number EF672685). Gene structure of the putative barramundi *MSTN-1 *gene (*LcMSTN-1*) was deduced by cross-comparing gDNA consensus sequence, the entire cDNA sequence from a single clone and the gilthead sea bream *MSTN-1 *gene [12]. Three exons were shown to be encoded by this gene in barramundi. Exon 1, exon 2 and exon 3 were 379, 371, 381 bp in length, whereas the enclosed introns were 412 and 810 bp, respectively. Distinctive sites were recognized: i) a typical start codon (ATG) in exon 1; ii) a stop codon (TGA) in exon 3; iii) distinctive splicing sites (GT...AG) at the exon-intron boundaries; iv) a putative TATA box 137 bp upstream from the ATG start codon (28 bp upstream the putative start transcription site); v) two putative E-boxes (CANNTG motif), 811 and 398 bp upstream from the ATG starting codon. Four single nucleotide polymorphisms were also identified in the 5' flanking region among barramundi from the seven locations: i) a T → G transversion (position 280); ii) a C → G transition (position 306); iii) a C → T transition (position 490); iv) a A → G transition (position 612). BLASTN search results showed that nucleotide sequence produced significant alignments (above 90%) with *MSTN-1 *gene sequences in Genbank, displaying extremely high identity with most perciformes. Within the gene sequence, higher rates of similarity were shared among coding regions, in particular exons 2 and 3.

**Figure 3:**
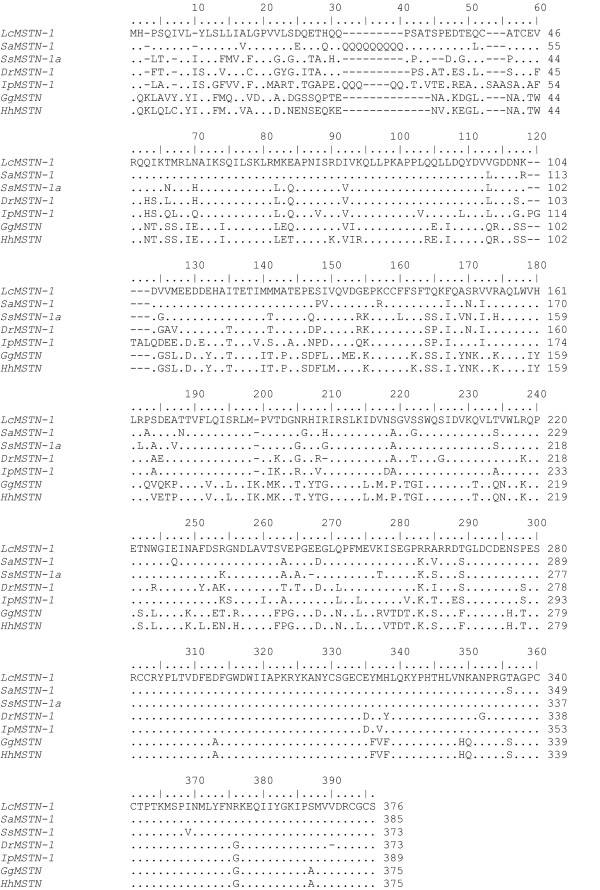
**Alignment of the barramundi *MSTN-1 *amino acid sequence with representative species of vertebrates**. Numbers on the right indicate amino acid positions. References and accession numbers are provided in appendix.

The translated amino acid sequence (376 aa) was used as a query for a BLASTP search, showing significant similarities with most vertebrate *MSTN *genes, including chicken and human (Fig. 3). Protein alignment between species representative of various classes of vertebrates showed that the amino acid sequence obtained in the current study coded for a protein with homologous structure to that observed in other vertebrate MSTN containing a pro-peptide domain (residues 41–258) and a bioactive domain (residues 282–376). One putative conserved proteolytic RXXR motif for the releasing of the mature peptide (matching with RARR, residues 264–267) was identified along with two putative conserved glycosylation sites (N) in the propeptide domain (residues 73 and 223) (Fig. 2). Finally, as for all vertebrate MSTN proteins, 13 conserved cysteines were found, nine of which were in the bioactive domain, a characteristic shared by all the TGF-β superfamily.

### Characterization of micro RNA target sites

**Figure 4:**
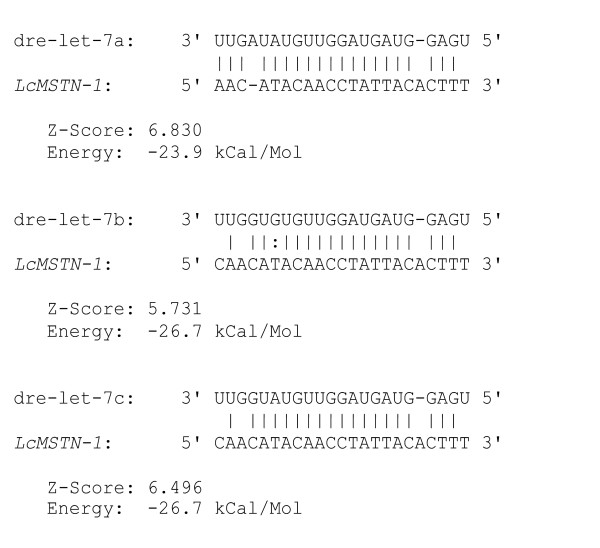
**Graphic representation of barramundi *MSTN-1 *(3'UTR) MiRNA target sites**. Double dot indicates G-U wobble bonds.

**Figure 5:**
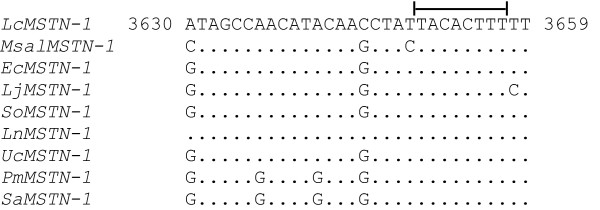
**Alignment of 3'UTR putative target site**. Bracket indicates the conserved site, matching with the critical 5' end of miRNA. Numbers refers to the nucleotide position relative to the barramundi *MSTN-1*. Abbreviations for species are provided in appendix.

Micro RNA (miRNA) target site analyses carried out on the barramundi 3'UTR genomic sequence revealed one putative site interacting with dre-let-7a, dre-let-7b and dre-let-7c, members of the let-7 miRNA family originally isolated in zebrafish (Fig. 4) [36]. A similar analysis of target sites in other teleost species indicated that all perciform *MSTN-1 *putatively interact with some members of the let-7 miRNA family. In particular, interaction of dre-let-7c was common to most species, with the only exception being gilthead seabream (data not shown). Atlantic salmon also displayed (with weak support value) a putative target site for dre-let-7d in a different 3'UTR region than the perciform counterpart, whereas no sites of interaction were found in zebrafish. An alignment of putative perciform interaction sites highlights 100% identity in the sequence complementary to the 5' critical region of the let-7 family (known as "seed") (5'-UGAGGUAG-3') (Fig. 5).

### Phylogenetic analyses

**Figure 6:**
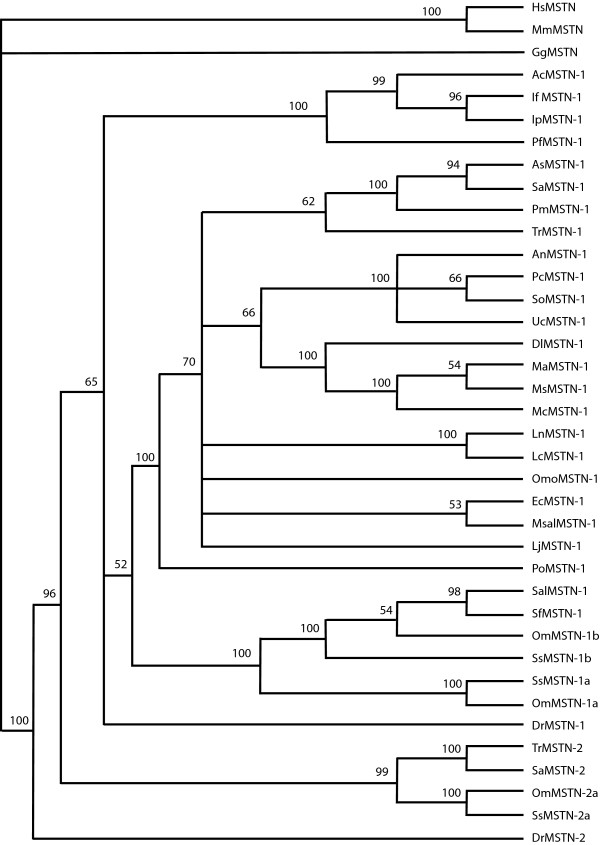
**Phylogenetic tree of teleosts *MSTN*-1 and -2 coding regions**. A maximum parsimony analysis was carried out using *HsMSTN*, *MmMSTN *and *GgMSTN *as outgroups. Numbers at the tree nodes represent percentage bootstrap support after 1000 replicates. Species abbreviations are shown in appendix. Tree topology and statistical support were similar for the NJ analysis (not shown).

**Figure 7:**
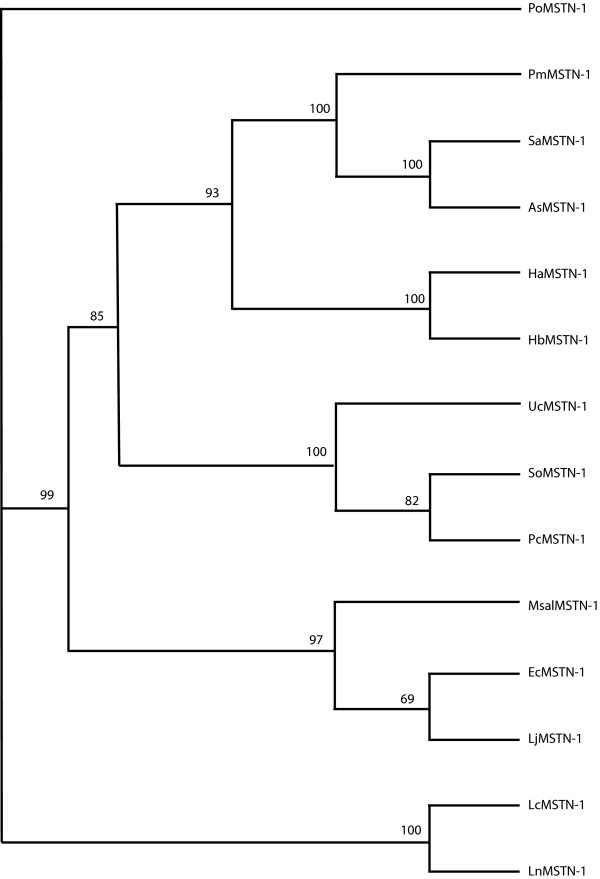
**Phylogenetic tree of perciformes whole *MSTN-1 *gene sequence**. No outgroup was used for the whole gene analysis. Numbers at the tree nodes represent percentage bootstrap support after 1000 replicates. Species abbreviations are shown in appendix. Tree topology and statistical support were similar for the NJ analysis (not shown).

Phylogenetic analyses of the *MSTN *gene and related sequences indicates that *LcMSTN-1 *clusters with high support (100%) with that of Nile perch *MSTN-1 *gene in the perciform clade. Phylogeny of *MSTN *coding regions shows a clear separation between teleost orders (siluriformes, salmoniformes, perciformes) and the second *MSTN *isoform, which are all grouped together with extremely high support (Fig. 6). The resulting tree also suggests that the sequence isolated in this study is the *LcMSTN-1 *isoform. The overall bootstrap values are reasonably high providing strong statistical support for the main structure of the tree, which is also supported by the known phylogenies of the represented species. However, the perciform branch was not fully resolved based purely on phylogeny of *MSTN *coding regions. Increased resolution of perciformes *MSTN-1 *evolutionary relationships arises from phylogenetic analyses achieved when the whole gene sequence was analysed (coding and intron regions) (Fig. 7).

### MSTN gene qualitative expression pattern

**Figure 8:**
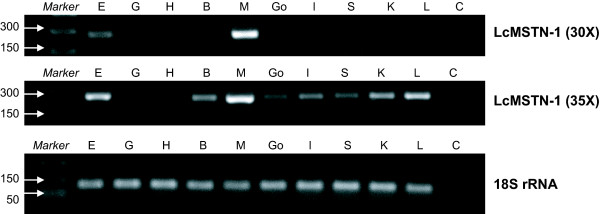
**Tissue-specific qualitative expression of the barramundi *MSTN-1 *gene**. Reverse transcriptase PCR was conducted for 30 cycles (30×) and 35 cycles (35×). Lanes are represented as follows: Marker; E, eye; G, gill; H, heart; B, brain; M, muscle; Go, gonad; I, intestine; S, spleen; K, kidney; L, liver; C, negative control. As a positive control of the RT-PCR reaction step, a 94 bp fragment was successfully amplified in 18S rRNA.

To further characterize the *LcMSTN-1 *gene, reverse transcriptase PCR (RT-PCR) was used to detect expression in eye, gill, heart, brain, muscle, gonad, intestine, spleen, kidney and liver (Fig. 8). The amplification products were identical in length and of the expected size. Cloning and sequencing the resulting 265 bp bands revealed that the fragments belonged to the *LcMSTN-1 *coding region. Transcripts were widely represented in each analyzed tissue by bands of variable intensities, suggesting that a possible differential expression pattern might occur among tissues. After 30 cycles, *LcMSTN-1 *transcripts were clearly visible in muscle and eye, intermediately detected in liver and kidney and weakly observed in brain, spleen and intestine. However, after 35 cycles, *LcMSTN-1 *transcripts were present in each tissue with very weak intensity in gills and heart. Amplification of RT control reactions showed no amplification products (data not shown).

## Discussion

In the present investigation we isolated and characterized the *MSTN-1 *gene from barramundi. The *LcMSTN-1 *contains three exons and two introns, as for all known vertebrate *MSTN*, and encodes for a 376 amino acid long peptide. Like other members of the TGF-β superfamily, MSTN is synthesized as a precursor peptide, which includes an N-terminal signal sequence, pro-peptide and bioactive domain [2]. Results of the BLASTP query show that *Lc*MSTN-1 protein structure is consistent with that previously reported by other authors [15,20]. In accordance with past studies, the peptide sequence is highly conserved in the C-terminal bioactive domain, where nine cysteine residues are present [1,2,20]. The nine conserved cysteines are involved in the formation of interchain disulphide-bonds, essential for the dimerization of the two MSTN subunits [37]. Two putative glycosylation sites were also identified in the pro-peptide domain. Glycosylation sites have also been demonstrated to be actively involved in the regulation of TGF-β superfamily bioactivity [38,39]. Finally, the RXXR motif (matching with RARR in *LcMSTN-1*) identified at residues 264–267 represents the putative cleavage site to release the processed mature peptide. The putative functional and structural roles associated with the above mentioned sites are corroborated by the extremely high identity maintained throughout the evolution of vertebrates.

With regard to the nucleotide sequence, *LcMSTN-1 *shares high similarity with most known perciform *MSTN-1*, particularly in the coding region. The 5' flanking region contains putative regulatory sites including a TATA box, located 28 bp upstream of the transcription starting point, and two E-boxes. The TATA box surrounding sequence (± 30 bp) is highly conserved across teleost species, sharing in this region 100% identity with other available sequence from perciformes. The E-box 1 is also found conserved in the same group of species that share the identical TATA box region. E-boxes have been shown to interact with the helix-loop-helix proteins of myogenic regulatory factors enhancing the expression of the *MSTN *gene in vertebrates, therefore representing interesting target sites for the screening of regulatory mutations [40].

A first screening of sequences from different Australian populations highlights that *LcMSTN-1 *is variable in the 5' flanking region, where four SNPs have been identified. These point mutations, however, did not appear to result in changes in putative functional sites. A lack of major polymorphisms in the coding region suggests that functional or loss-of-function mutations might be rarer in fish than in higher vertebrates [28,31]. However, the number of individuals targeted in this study did not provide enough elements for robust conclusions. As wild fish are continuously utilized in hatcheries for the production of barramundi larvae, it is essential in future studies to screen a larger number of individuals/populations and understand the spatial genetic variability of *LcMSTN-1*. A better understanding of *LcMSTN-1 *variability will allow selection of appropriate brood-stocks in an experimental *MSTN*-based selective breeding program. Finally, it is notable that repeated motifs of significant length were not found in non-coding regions. In particular, from sequence alignment emerges that intron 2 contains a (CA)_n_-SSR (Simple Sequence Repeat) which is observed to be extremely conserved across perciformes and salmonids, once again suggesting a possible functional role (i.e. gene expression regulation [32]). In barramundi the CA-microsatellite is reduced to five repeated units.

The central finding of this study is the identification of a miRNA target in the 3'UTR. MiRNA are small non-coding RNA (~22 nucleotide) that can actively regulate the expression of several genes at the post-transcriptional level by physical interaction with complementary mRNA sequences [41]. Sites presenting perfect complementarity at position 2–8 of the 5' end of the miRNA are likely to be targeted by the miRNA itself [42], but sites with one mismatch in the target sequence have also been considered as seeds [43]. Herein, a site located in the 3'UTR of *LcMSTN-1 *and highly conserved across perciformes represents a putative target for the let-7 miRNA family. To support this finding, members of the let-7 family have been predicted, via evolutionary conservation, to interfere with the TGF-β signalling pathway [43]. To highlight the importance of gene expression regulation at the post-transcriptional level, a recent study has described a polymorphism in the 3'UTR of the sheep *MSTN *gene that generates an illegitimate new target site for two miRNAs [44]. This point mutation was statistically associated with an increased hypertrophy of muscle fibres, therefore defining a new class of regulatory mutations. The homologous site of interaction, herein described for the first time in finfish, represents a possible valuable target for the identification of fast growing haplotypes. Further, miRNAs may also play a pivotal developmental role inhibiting the translation of *MSTN *in lower vertebrates in a tissue-specific manner.

It has been well established that the piscine *MSTN *gene evolved subject to different events of duplication and in some cases tissue-specific differential expression of *MSTN-*1 and *MSTN-2 *was detected [9,11,22,23,45]. Loss of physiological function might have evolved in parallel to *MSTN *duplication events as, for example, in the case of salmonids where different *MSTN *isoforms are specifically expressed in distinct tissues [9,11,45]. However, it is possible that miRNAs have a crucial role in regulating the expression of piscine *MSTN *genes. The latter hypothesis is strongly supported by miRNA differential expression detected in teleost fishes [46] and might also explain the loss of the miRNA target site observed in salmonids compared to the sister clade of perciformes. Therefore, it would be useful in future studies to target the miRNA pathway as a source of variation for the identification of fast growing mutants and the improvement of finfish productivity. In fact, although mutations occurring at *MSTN *miRNA target sites have been shown to affect muscularity in higher vertebrates [44], the post-transcriptional regulatory pathway has not been targeted in finfish to date.

The phylogeny of the *MSTN *gene has been widely assessed by previous authors and is largely consistent with the current study [12,20,21,47]. However, former investigations have been carried out on protein sequence of a limited number of species [12,21]. Thanks to recent intensive submissions of piscine *MSTN *genes to public databases, we present the first attempt to assess the utility of *MSTN *introns as a phylogenetic nuclear marker. In accordance with the tree presented by Ye *et al*. [20], phylogeny of coding regions was unable to produce a fully resolved perciform clade. On the contrary, when coding regions were analyzed in conjunction with introns a better picture of perciformes evolutionary relationships emerged. In particular, largemouth sea bass, Japanese sea perch and orange spotted grouper were clustered together, with high bootstrap values supporting the monophyly of this clade. The resolution within the perciformes using the complete *MSTN-1 *gene is consistent with that of Smith *et al *[48] based on 4036 bp of mitochondrial and nuclear sequence, demonstrating that *MSTN *also provides strong phylogenetic signal and has utility as a marker for future broader piscine phylogeny studies.

The principal biological activity of MSTN is to negatively regulate growth of muscle fibres [2]. The suppression of hyperplasic and hypertrophic growth of somites triggered by MSTN has been experimentally demonstrated in vertebrates, where the inactivation of protein synthesis leads to an extraordinary increase of muscle mass both in fish and mammals [2,6]. However, in mammals MSTN physiological activity seems to be restricted to skeletal muscle and cardiomyocytes and major loss-of-function natural mutations have been identified, causing giant phenotypes without any other detectable side effects [3,28,49]. On the contrary, previous investigations are contrasting with regard to *MSTN *expression in spleen, kidney, liver and intestine of teleost fishes. In particular, tissues that showed no *MSTN *transcript detection included kidney in Japanese sea perch [20], spleen in croceine croaker [21], gonad, liver and spleen in gilthead seabream [12] and adipose tissue, liver, spleen, intestine and kidney in shi drum [10]. In the present study, *MSTN *transcripts were expressed at some level in each target tissue examined. However, in gills and heart the expression was so weak that 35 amplification cycles were needed to visualize a PCR product. These results do demonstrate, however, that in adult barramundi at least, *MSTN *is widely expressed in many tissues at the same time.

Although one aim of the present investigation was simply to determine the qualitative tissue-specific expression of the *MSTN *gene and quantitative conclusion without verification from quantitative PCR might be hazardous, it was clearly evident that *LcMSTN-1 *transcripts were more highly expressed in barramundi skeletal muscle than in other tissues. This is a similar finding to other fish studies where *MSTN *expression has been shown to be highest in skeletal muscle, and sometimes, one or other tissues like brain, eye or liver [9,10,16,20]. Unlike many other studies, however, we observed some level of simultaneous *LcMSTN-1 *expression in skeletal muscle, eye, gill, heart, brain, muscle, gonad, intestine, spleen, kidney and liver. The fact that *LcMSTN-1 *transcripts were not found to be simultaneously expressed in such a wide range of non-muscle tissues in other fish might have depended on several factors including methodology (i.e. number of amplification cycles – in tissues where the transcripts are very weak a difference of five cycles is significant), tissue preservation (RNA degrades relatively quickly) or age effects on expression (*MSTN *expression intensity is significantly correlated with developmental stages [15]).

## Conclusion

The *LcMSTN-1 *gene was successfully isolated and characterized. The gene displays high sequence identity and equal organization both with other teleost fish and mammalian counterparts. Putative functional sites have been recognized in *LcMSTN-1*, either in the translated protein and nucleotide sequence. *LcMSTN-1 *transcripts are ubiquitously expressed in several tissues with greater intensity in skeletal muscles. The high similarity observed across teleost, mammalian and avian species indicates that the main biological function of MSTN (muscle growth suppressor) has been evolutionary conserved to some extent, although some physiological activities might have been lost/acquired during the evolution of vertebrates. However, the successful association between *MSTN *gene variation and growth traits observed in higher vertebrates corroborates the importance that the *MSTN *gene might have for the improvement of aquaculture finfish production and encourages further investigations. Furthermore, a putative miRNA site of interaction identified in this study and localized in the 3'UTR suggests a possible new direction to be undertaken for the improvement of finfish productivity.

## Methods

### Sample collection

Three one-year-old barramundi were collected from a local barramundi farm. Fish were euthanized by diluting 1 ml clove oil per litre of water. Brain, muscle, liver, intestine, heart, eye, spleen, gills, gonads, and kidney were dissected, immediately preserved in liquid nitrogen and subsequently stored at -80°C until further processing. Fin clips were also removed and preserved in 100% ethanol. Furthermore, fin clips from seven wild-caught barramundi were collected from seven separated Australian locations (Ashburton River (Western Australia – WA), De Grey River (WA), Roebuck Bay (WA), Ord River (WA), Daly River (Northern Territory), Gulf of Carpentaria (Queensland – QLD), Boyle River (QLD)) to provide an opportunity for maximum levels of genetic variability and preserved in 100% ethanol.

### Cloning and characterization of the MSTN gene

Total genomic DNA (gDNA) was extracted from barramundi fin clips by digestion in CTAB buffer with 20 mg/ml proteinase K for 1 hour at 60°C, followed by a phenol:chloroform:isoamyl alcohol purification protocol [50]. Genomic DNA was quantified by comparison to DNA concentration standards after 0.8% agarose gel electrophoresis using the ImageJ 1.33 software package (Wayne Rasband, National Institutes of Health, MA) and re-suspended in ddH2O to a concentration of 10 ng/μl.

Twelve specific oligonucleotides were designed based on conserved regions of known *MSTN-1 *sequences in perciformes (Table 1). Four pairs of primers, Myo-up1/Jcu-R, Myo-up2/Myo-L1, Myo-up3/Myo-L3, Myo-up5/Myo-L5 were designed. For the amplification of 5' and 3' UTRs (untranslated regions) two pairs of primers, Myo-pro/Myo-L8 and Myo-up8/Myo-utr (Table 1), were designed. PCR reactions were carried out in a final volume of 50 μl using the following final concentration of reagents: 1 × of 10× PCR buffer (Qiagen), 2 mM of MgCl_2_, 0.4 mM of dNTP mix, 0.4 μM of each primer, 0.75 units of *Taq *polymerase (Qiagen) and 10–20 ng of gDNA. The amplification cycle, performed in a MJ research thermocycler, consisted of 2 min pre-denaturation at 94°C followed by 32 cycles of denaturation at 94°C for 45 sec, annealing at specific temperature (Table 1) for 45 sec and elongation at 72°C for 1 min, followed by 10 min final extension at 72°C. The PCR fragments of expected size obtained from these amplifications were separated on 1.5% agarose gels, purified and cloned into a pGEM-T Easy Vector System (Promega, Madison, WI). Clones containing the insert of expected size were isolated and sequenced in both directions using T7 and SP6 universal primers. Fragment sequences were analysed and spliced in to a single consensus sequence using Sequencher version 4.2 (GeneCode Corp) and visually analysed for polymorphisms. In addition, the whole cDNA was sequenced in both directions from three single clones and compared to the gDNA sequence as a control.

**Table 1:** Primer names, sequences and annealing temperatures used for the characterization of barramundi *MSTN-1 *gene

Primer name	Sequence (5'-3')	Annealing temperature (°C)
Myo-up1	ATG CAT CYG TCT CAG ATT GTG [21]	52
Myo-up2	ATG AGC AYG CCA TCA CGG AGA	52
Myo-up3	CAA CTG GGG CAT CSA GAT TAA C	52
Myo-up5	GCT ACA AGG CCA ACT ATT GC	52
Myo-up8	ACA TAC AAC CTA TTA CAC	45
Myo-pro	CCA CAC GAT GGC RCT RTC	55
Jcu-R	GTT CAG TGG CCA TCA TCA T	52
Myo-L1	TTT CCC CTC GAA TCG AAG GC	52
Myo-L3	GCA ATA GTT GGC CTT GTA GCG	52
Myo-L5	TGA GGA TTC CTG GTT TCA CTC	52
Myo-L8	TGC TGG TGC GTT TCT TGG TC	55
Myo-Utr	GGC ATA TGT ATA CAA TAC	45
18S-F	TGGTTAATTCCGATAACGAACGA	54-45 step-down
18S-R	CGCCACCTGTCCCTCTAAGAA	54-45 step-down

### Genomic sequence: nucleotide analysis

The resulting *LcMSTN-1 *consensus nucleotide and amino acid sequences were first compared with those available from the Genbank public database using BLASTN and BLASTP, respectively [51]. The intron-exon boundaries were deduced by alignment with cDNA sequence amplified using the Myo-up1/Myo-L3 and Myo-up3/Myo-L5 primer pairs (Table 1). Translated amino acid sequences were aligned for analysis of putative conserved functional residues by ClustalW using MEGA 3.1 [52]. Putative full-transcripts sequences, starting and stop codon and other functional sites where deduced by comparison with known *MSTN *sequences.

### Micro RNA target sites identification

To find potential miRNA target sites, a further analysis of non-coding regions was carried out using the miRanda software package [53], using default settings and SHUFFLE mode ON (1000 random shuffled analysis). The entire collection of *Danio rerio *miRNA available from a public non-coding RNA database [52,54-56] was used as a query and run against *LcMSTN-1 *3'UTR. Output parameters considered for the scope of this investigation were: i) score; ii) energy and iii) z-value (index of rudimental statistical test for the reliability of results) [53]. Threshold score and energy were set at 100 and -19 kcal/mol respectively. MiRNA showing a putative interaction with *LcMSTN-1 *3'UTR were then tested on representative teleosts species using default thresholds and parameters. Finally, presumed target sites of interactions were visually confirmed by screening sequence site conservation between relatively close species.

### Phylogenetic analysis

Phylogenetic analysis of evolutionary relationship between *LcMSTN-1 *and other vertebrates was performed using two optimality criteria. Firstly, a maximum parsimony analysis with tree-bisection-reconnection (TBR) branch-swapping and gaps treated as missing characters was undertaken in PAUP version 4.08 [57]. Secondly, a neighbour-joining (NJ) tree was constructed using the Jukes-Cantor model of sequence evolution in MEGA 3.1 [52]. Support for both types of trees was obtained by bootstrapping data (1000 replicates). Sequences were rooted using *Homo sapiens*, *Mus musculus *and *Gallus gallus *as outgroups.

### Expression analysis: RNA extraction, complementary DNA synthesis and Reverse Transcriptase PCR

Total RNA was extracted from preserved barramundi tissues with Trizol reagent (Invitrogen, Carlsbad, CA). RNA quality and DNA contamination were verified by 1.5% agarose gel electrophoresis. RNA concentration and purity was checked on a spectrophotometer using 230/260 and 260/280 nm wavelengths. Nucleic acid samples were diluted in ddH_2_O to a concentration of 100 ng/μl. First strand complementary DNA (cDNA) was synthesized from the same amount of total RNA (1 μg) for each sample using SuperScript III Reverse Transcriptase enzyme (SS-III) (Invitrogen, Carlsbad, CA). A mixture of (T)25 oligos and random hexamers were used as primers for the reverse transcriptase reaction. A further reaction was carried out without the SS-III (-RT) enzyme as a control. 2 μl of the resulting cDNA and -RT control were used as templates for the RT-PCR. RT-PCR reactions were prepared in a final volume of 25 μl with primers Myo-up3 and Myo-L3, designed to encompass intron 2 in order to eliminate potential positive results due to genomic contamination. Concentrations of reagents were maintained consistent with the ones previously used for gDNA amplifications. To confirm the specificity of the amplification, fragments of expected size (approximately 270 bp) were cloned and sequenced. In RT-PCR, 18S rRNA (18S-F and 18S-R primers, Table 1) were used as a control to test the quality of the reverse transcribed RNA. A 55 to 48°C step-down program was used for the 18S rRNA amplification reaction. The step-down program consisted of: 94°C × 3 min (pre-denaturation); 4× (94°C × 30 sec + 55°C × 40 sec + 72°C × 1 min) + 4× (94°C × 30 sec + 50°C × 40 sec + 72°C × 1 min) + 20× (94°C × 30 sec + 48°C × 40 sec + 72°C × 1 min); 72°C × 10 min (final extension).

## Authors' contributions

CDS and DRJ conceived the intellectual design of the project and co-wrote the manuscript. Laboratory work was undertook by CDS. CSK and BSE formed part of the supervisory team and provided intellectual input into experimental design, statistical analyzes and laboratory procedures. All authors read and approved the final manuscript.

## Appendix

List of abbreviations and Genbank accession number of fish species used in alignments and phylogenetic analyses (see Table 2).

**Table 2:** 

*Abbreviation*	*Species*	*Common name*	*Genebank nucleotide accession no*
*AsMSTN-1*	*Acanthopagrus schlegelii*	Black sea bream	DQ251470
*EcMSTN-1*	*Epinephelus coioides*	Orange spotted grouper	DQ493889
*LjMSTN-1*	*Lateolabrax japonicus*	Japanese sea perch	AY965685
*MsalMSTN-1*	*Micropterus salmoides*	Largemouth bass	EF071854
*PmMSTN-1*	*Pagrus major*	Red seabream	AY965686
*PcMSTN-1*	*Pseudosciaena crocea*	Croceine croacker	AY842934
*SoMSTN-1*	*Sciaenops ocellatus*	Red drum	DQ855526
*UcMSTN-1*	*Umbrina cirrosa*	Shi drum	AF316882
*SaMSTN-1*	*Sparus aurata*	Gilthead sea bream	AF258447
*SaMSTN-2*	*Sparus aurata*	Gilthead sea bream	AY046314
*AnMSTN-1*	*Atractoscion nobilis*	White weakfish	AY966401
*DlMSTN-1*	*Dicentrarchus labrax*	European sea bass	AY839106
*MaMSTN-1*	*Morone americana*	White perch	AF290911
*McMSTN-1*	*Morone chrysops*	White bass	AF197194
*MsMSTN-1*	*Morone saxatilis*	Striped bass	AF290910
*OmoMSTN-1*	*Oreochromis mossambicus*	Mozambique tilapia	AF197193
*LcMSTN-1*	*Lates calcarifer*	Barramundi	EF672685
*HaMSTN-1*	*Harpagifer antarcticus*	Antarctic spiny plunderfish	AY926479
*HbMSTN-1*	*Harpagifer bispinis*	Magellan plunderfish	AY953943
*LnMSTN-1*	*Lates niloticus*	Nile perch	EF681885
*SsMSTN-1a*	*Salmo salar*	Atlantic salmon	EF392862
*SsMSTN-1b*	*Salmo salar*	Atlantic salmon	AJ316006
*SsMSTN-2a*	*Salmo salar*	Atlantic salmon	EF392863
*OmMSTN-1a*	*Oncorhynchus mykiss*	Rainbow trout	DQ136028
*OmMSTN-1b*	*Oncorhynchus mykiss*	Rainbow trout	DQ138300
*OmMSTN-2a*	*Oncorhynchus mykiss*	Rainbow trout	DQ138301
*SalMSTN-1*	*Salvelinus alpinus*	Arctic char	AJ829532
*SfMSTN-1*	*Salvelinus frontinalis*	Brook trout	AF247650
*DrMSTN-1*	*Danio rerio*	Zebrafish	AY323521
*D-MSTN-2*	*Danio rerio*	Zebrafish	AY693972
*TrMSTN-1*	*Takifugu rubripes*	Fugu	AY445322
*TrMSTN-2*	*Takifugu rubripes*	Fugu	AY445321
*PoMSTN-1*	*Paralichthys olivaceus*	Bastard halibut	DQ997779
*IpMSTN-1*	*Ictalurus punctatus*	Channel catfish	AF396747
*AcMSTN-1*	*Ameiurus catus*	White catfish	AY540994
*IfMSTN-1*	*Ictalurus furcatus*	Blue catfish	AY540992
*PfMSTN-1*	*Pseudobagrus fulvidraco -*	Yellow catfish	DQ767966
*MmMSTN*	*Mus musculus*	Mouse	BC103678
*HsMSTN*	*Homo sapiens*	Human	BC074757
*GgMSTN*	*Gallus gallus*	Chicken	AF019621

## Supplementary Material

Additional file 1 Additional file 1. Click here for file
